# Estimated pulse wave velocity and progression to advanced cardiovascular–kidney–metabolic syndrome: a population-based longitudinal study with supportive external cross-sectional evidence

**DOI:** 10.3389/fcvm.2026.1875961

**Published:** 2026-07-08

**Authors:** Wenjiao Zhang, Wenbo Yang, Yirui Xu, Heze Fan, Hao Yuan, Qi Kang, Shumei Zhang, Peiyao Zhang, Eerwei Hu, Xueying Feng, Lijun Wang, Zuyi Yuan, Juan Zhou

**Affiliations:** 1 Department of Cardiovascular Medicine, The First Affiliated Hospital of Xi'an Jiaotong University, Xi'an, Shaanxi, China; 2 Key Laboratory of Environment and Genes Related to Diseases, Ministry of Education, Xi'an Jiaotong University, Xi'an, Shaanxi, China

**Keywords:** arterial stiffness, cardiovascular–kidney–metabolic syndrome, China health and retirement longitudinal study, estimated pulse wave velocity, longitudinal study

## Abstract

**Background:**

Cardiovascular–kidney–metabolic (CKM) syndrome captures the interconnected burden of metabolic risk factors, chronic kidney disease, and cardiovascular disease. Evidence remains limited regarding simple and clinically accessible markers that may help identify individuals at risk of progression to advanced CKM syndrome. We evaluated whether estimated pulse wave velocity (ePWV), an easily calculated marker of arterial stiffness, was associated with progression to advanced CKM syndrome.

**Methods:**

We analyzed longitudinal data from the China Health and Retirement Longitudinal Study, with baseline assessment in 2011 and follow-up assessments in 2013 and 2015. Multivariable logistic regression examined associations of baseline ePWV, longitudinal changes in ePWV, and ePWV trajectories with progression to advanced CKM syndrome. Subgroup, inverse probability weighting, restricted cubic spline, and receiver operating characteristic analyses were performed. We also assessed cross-sectional associations in an independent hospital-based cohort.

**Results:**

The longitudinal analysis included 5,810 participants. In fully adjusted models, higher baseline ePWV was associated with greater odds of progression to advanced CKM syndrome. Compared with participants in the lowest ePWV tertile, those in the highest tertile had significantly higher odds of CKM progression [odds ratio [OR] = 2.630; 95% confidence interval [CI]: 2.004–3.451; *P* for trend <0.001]. The association was generally consistent across subgroups, and inverse probability weighting analyses supported the robustness of the findings after accounting for loss to follow-up. Each 1 m/s increase in change in ePWV was associated with 115.3% higher odds of CKM progression (OR = 2.153; 95% CI: 1.960–2.364), and a persistently high ePWV trajectory was also associated with greater odds of CKM progression. Restricted cubic spline analysis showed a nonlinear association, and baseline ePWV showed moderate discriminatory performance. The association between higher ePWV and advanced CKM syndrome was also supported by the independent hospital-based cross-sectional cohort.

**Conclusions:**

Higher baseline ePWV, greater longitudinal increases in ePWV, and a persistently higher ePWV trajectory were associated with progression to advanced CKM syndrome. ePWV may serve as a convenient adjunctive marker for early CKM risk stratification, while its interpretation should consider its dependence on age and blood pressure.

## 1 Introduction

The American Heart Association (AHA) introduced the cardiovascular–kidney–metabolic (CKM) syndrome framework in 2023, defining CKM syndrome as an integrated clinical and pathophysiological construct that reflects the complex interplay among obesity, metabolic disorders, chronic kidney disease (CKD), diabetes, and cardiovascular dysfunction (1, 2). Epidemiological evidence indicates that CKM syndrome is highly prevalent, with approximately 90% of U.S. adults meeting criteria for stage 1 or higher and about 15% classified as having advanced CKM stages (3). Given this substantial population burden, simple and accessible markers that can identify individuals at risk of CKM progression may support earlier risk stratification and prevention-oriented public health strategies.

Arterial stiffness is increasingly recognized as a vascular mechanism linking metabolic abnormalities, renal dysfunction, and cardiovascular disease (CVD) (4–6). Although pulse wave velocity is considered the reference standard for assessing arterial stiffness (7), its large-scale implementation is limited by the need for specialized devices and standardized technical procedures. Estimated pulse wave velocity (ePWV) has emerged as a practical alternative that can be calculated from age and mean blood pressure (MBP). In the study by Greve et al., ePWV showed a moderate association with measured carotid–femoral pulse wave velocity, with reported coefficients of determination ranging from 0.268 to 0.448 (8). Among participants in the Danish 10-year follow-up cohort of the World Health Organization Multinational Monitoring of Trends and Determinants in Cardiovascular Disease Project, Bland–Altman analysis demonstrated a relative difference of −0.3% between ePWV and measured carotid–femoral pulse wave velocity, with a reported 95% confidence interval (CI) ranging from −15% to +17% (8). Despite this variability between the two measures, elevated ePWV has been associated with adverse cardiovascular outcomes (9, 10), metabolic disorders (11), and CKD (12), suggesting that it may reflect the shared vascular burden underlying CKM syndrome.

Previous studies have linked ePWV to mortality and cardiovascular events (13, 14), but most have focused on individual outcomes, such as incident heart disease, stroke, or composite CVD (9, 15, 16). Evidence remains limited on whether ePWV is associated with progression to advanced CKM syndrome under the integrated CKM staging framework. Therefore, using data from the nationally representative China Health and Retirement Longitudinal Study (CHARLS), we investigated the association between ePWV and progression to advanced CKM syndrome. To assess the robustness of the association, we further conducted a supportive cross-sectional analysis in an independent hospital-based cohort. By integrating longitudinal evidence from the population-based CHARLS cohort with supportive cross-sectional evidence from the external hospital-based cohort, this study aimed to evaluate the potential utility of ePWV as a simple and scalable marker for early CKM risk stratification.

## 2 Materials and methods

### 2.1 Data source and study population

**Figure 1:**
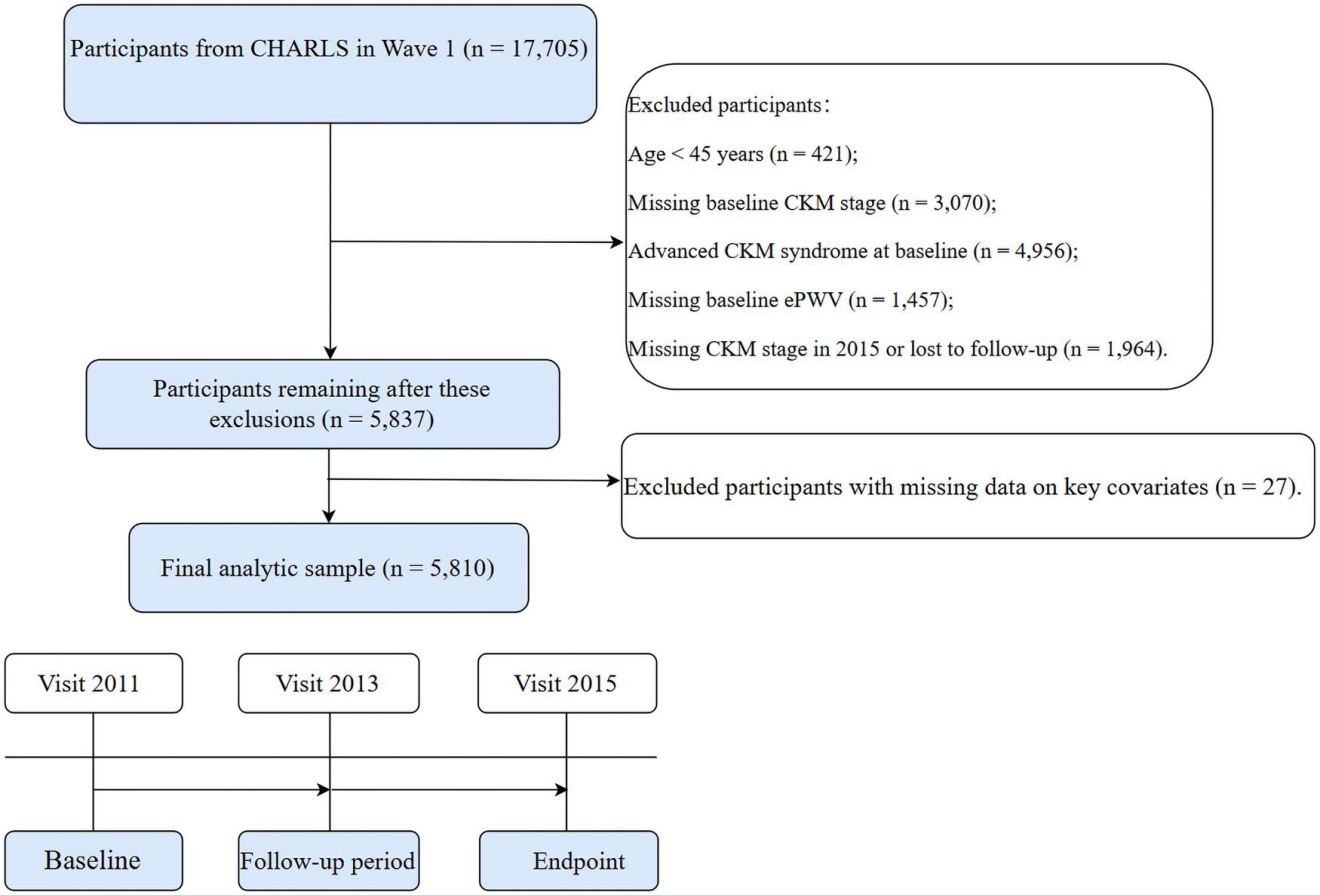
Flowchart of participant selection from the CHARLS cohort for the longitudinal analysis. CHARLS, China health and retirement longitudinal study; CKM, cardiovascular–kidney–metabolic; ePWV, estimated pulse wave velocity.

CHARLS is a nationally representative longitudinal survey conducted in 28 provinces across mainland China. The baseline survey was conducted in 2011, with follow-up waves in 2013 and 2015. A total of 17,705 participants were enrolled at baseline. Participants were excluded for the following reasons: age <45 years at baseline (*n* = 421), missing information required to determine baseline CKM stage (*n* = 3,070), advanced CKM syndrome (stages 3–4) at baseline (*n* = 4,956), missing baseline ePWV data (*n* = 1,457), missing CKM stage data in 2015 or loss to follow-up at Wave 3 (*n* = 1,964), and missing data on key covariates (*n* = 27). The final longitudinal analytic sample included 5,810 participants. The participant selection process is shown in Figure 1.

To further assess the robustness and consistency of the CHARLS findings, we included an independent hospital-based cross-sectional cohort. Clinical data were obtained from individuals who underwent routine health examinations at the Health Examination Center, the First Affiliated Hospital of Xi'an Jiaotong University, Xi'an, China. This single-center, hospital-based cross-sectional cohort included participants examined between May 1 and May 31, 2024. A total of 4,423 individuals were initially identified. Participants were excluded if they were aged <45 years or had missing age information (*n* = 2,369), had missing ePWV data (*n* = 206), or had missing CKM stage data (*n* = 252). After these exclusions, 1,596 participants were included in the supportive cross-sectional analysis. The participant selection process is summarized in Supplementary Figure S1.

### 2.2 Definition of ePWV

In the CHARLS cohort, blood pressure was measured twice, and the mean of the two readings was used to define systolic blood pressure (SBP) and diastolic blood pressure (DBP). In the hospital-based cohort, SBP and DBP were obtained from routine health examination records. MBP was calculated as DBP + 0.4 × (SBP−DBP), and ePWV was subsequently derived from age and MBP using the established methodology (8, 17): ePWV = 9.587 − 0.402 × age + 0.00456 × age^2^ − 0.00002621 × age^2^ × MBP + 0.003176 × age ×MBP − 0.01832 × MBP.

### 2.3 Definition of CKM syndrome stages

CKM syndrome stages were defined according to the AHA Presidential Advisory on CKM syndrome (1), following the classification approach described by Aggarwal and colleagues (3). In brief, Stage 0 was defined as the absence of CKM risk factors, including normal adiposity, normoglycemia, normotension, a normal lipid profile, and no evidence of CKD or CVD. Stage 1 represented early metabolic risk, including elevated body mass index, elevated waist circumference, or prediabetes. Stage 2 was defined by the presence of metabolic risk factors or moderate CKD risk, including elevated triglycerides, hypertension, diabetes, metabolic syndrome, or moderate-risk CKD. Stage 3 indicated subclinical or high-risk CKM syndrome, including very-high-risk CKD, defined as an estimated glomerular filtration rate of <30 mL/min/1.73 m^2^, or a high predicted 10-year CVD risk. The 10-year CVD risk was estimated using the Framingham General Cardiovascular Risk Score equations (18). Stage 4 indicated self-reported established CVD, including coronary heart disease, angina, heart attack, heart failure, or stroke. Participants were classified according to the highest CKM stage for which they met the criteria. Advanced CKM syndrome was defined as stages 3–4, whereas stages 0–2 were considered non-advanced CKM syndrome. Detailed operational definitions and thresholds for each CKM stage are provided in Supplementary Table S1.

### 2.4 Covariates

Covariates included age, sex, education level, marital status, smoking status, and alcohol status. In the CHARLS cohort, multivariable logistic regression models were adjusted for these covariates. In the hospital-based cross-sectional cohort, models were adjusted for age and sex because other covariates were not consistently available.

### 2.5 Statistical analysis

Continuous variables are presented as mean ± standard deviation, and categorical variables are summarized as counts and percentages. Between-group differences were assessed using Student's *t* test for continuous variables and the chi-square test or Fisher's exact test for categorical variables. Logistic regression models were used to examine the association between baseline ePWV and progression to advanced CKM syndrome (stages 3–4) in the longitudinal CHARLS cohort. ePWV was primarily analyzed according to tertiles. Additional analyses modeled ePWV as a continuous variable (per 1 m/s increase), by quartiles, and as a binary variable based on the highest quartile (Q4). Odds ratios (ORs) and 95% CIs were estimated using logistic regression. Linear trends across tertiles were evaluated by modeling the ordinal exposure variable as a continuous term. Three multivariable models were constructed. Model 1 was adjusted for age and sex. Model 2 was additionally adjusted for education level, marital status, smoking status, and alcohol status. Model 3 was further adjusted for baseline CKM stage. Subgroup analyses were performed to assess the consistency of the association between ePWV and CKM progression across strata defined by age, sex, alcohol status, history of hypertension, and history of diabetes. Potential bias due to missing 2015 CKM data or loss to follow-up was assessed using an inverse probability weighting sensitivity analysis. Stabilized weights were estimated using baseline covariates, truncated at the 1st and 99th percentiles, and then applied in weighted logistic regression models. The association between change in ePWV and progression to advanced CKM syndrome was also examined. Change in ePWV was defined as the follow-up ePWV value in 2015 minus the baseline value in 2011. Latent class mixed models with one to four classes were fitted to identify ePWV trajectories from 2011 to 2015. Based on model fit, classification diagnostics, parsimony, and clinical interpretability, a two-class model was selected, and its association with CKM progression was evaluated using multivariable logistic regression. Receiver operating characteristic (ROC) analysis assessed the discrimination of baseline ePWV for CKM progression, while changes in area under the curve (AUC), net reclassification improvement, and integrated discrimination improvement evaluated its incremental value beyond traditional CKM risk factors. Restricted cubic spline (RCS) logistic regression with four knots was used to assess potential nonlinear associations between ePWV and CKM progression.

In the hospital-based cross-sectional cohort, ePWV was primarily analyzed as a continuous variable per 1 m/s increase and according to tertiles. Supplementary analyses further examined ePWV using quartile-based categories and a binary classification. Logistic regression models were used to assess the association between ePWV and advanced CKM syndrome. Model 1 was unadjusted, and Model 2 was adjusted for age and sex. All statistical analyses were performed using R software version 4.3.2, and a two-sided *P*-value <0.05 was considered statistically significant.

## 3 Results

### 3.1 Baseline characteristics according to progression to advanced CKM syndrome

A total of 5,810 participants with baseline CKM stages 0–2 were included in the longitudinal analysis. The mean age was 56.9 ± 8.4 years, and 3,777 participants (65%) were female. During follow-up from 2011 to 2015, 941 participants progressed to advanced CKM syndrome. Compared with participants who did not progress, those who progressed were older (62.2 ± 8.7 vs. 55.9 ± 8.0 years, *P* < 0.001) and more likely to be male (46% vs. 33%, *P* < 0.001). They also had higher SBP (136.2 ± 22.2 vs. 127.0 ± 18.8 mmHg, *P* < 0.001) and DBP (78.3 ± 13.2 vs. 75.5 ± 11.6 mmHg, *P* < 0.001). Significant differences were also observed in marital status and baseline CKM stage distribution (both *P* < 0.001), whereas education level, smoking status, and alcohol status did not differ significantly. Detailed baseline characteristics are shown in Table 1. Participants excluded because of missing baseline CKM staging information were broadly similar in age based on standardized difference but differed in sex distribution and multiple cardiometabolic characteristics (Supplementary Table S2).

**Table 1:** Baseline characteristics of participants in the CHARLS cohort according to progression to advanced CKM syndrome.

Characteristic	Overall (*N* = 5,810)	No CKM progression (*N* = 4,869)	CKM Progression (*N* = 941)	*P*-value
Age, years	56.9 ± 8.4	55.9 ± 8.0	62.2 ± 8.7	<0.001
Sex, *n* (%)				<0.001
Male	2,033 (35%)	1,597 (33%)	436 (46%)	
Female	3,777 (65%)	3,272 (67%)	505 (54%)	
Education level, *n* (%)				0.058
Primary school or below	3,940 (68%)	3,275 (67%)	665 (71%)	
Middle school	1,281 (22%)	1,101 (23%)	180 (19%)	
High school or above	589 (10%)	493 (10%)	96 (10%)	
Marital status, *n* (%)				<0.001
Married	5,191 (89%)	4,400 (90%)	791 (84%)	
Previously married	591 (10%)	446 (9.2%)	145 (15%)	
Never married	28 (0.5%)	23 (0.5%)	5 (0.5%)	
Smoking status, *n* (%)				0.106
Non-smoker	4,653 (80%)	3,918 (80%)	735 (78%)	
Current smoker	1,157 (20%)	951 (20%)	206 (22%)	
Alcohol status, *n* (%)				0.894
Non-drinker	4,101 (71%)	3,439 (71%)	662 (70%)	
Drinker	1,709 (29%)	1,430 (29%)	279 (30%)	
SBP, mmHg	128.5 ± 19.7	127.0 ± 18.8	136.2 ± 22.2	<0.001
DBP, mmHg	75.9 ± 11.9	75.5 ± 11.6	78.3 ± 13.2	<0.001
Baseline CKM stage, *n* (%)				<0.001
Stage 0	445 (7.7%)	406 (8.3%)	39 (4.1%)	
Stage 1	1,869 (32%)	1,680 (35%)	189 (20%)	
Stage 2	3,496 (60%)	2,783 (57%)	713 (76%)	

CHARLS, China health and retirement longitudinal study; CKM, cardiovascular–kidney–metabolic; SBP, systolic blood pressure; DBP, diastolic blood pressure.

### 3.2 Association between ePWV and progression to advanced CKM syndrome

Baseline ePWV was categorized into tertiles. Logistic regression analysis showed that higher ePWV categories were associated with greater odds of progression to advanced CKM syndrome (Table 2). In Model 1, participants in the second and third tertiles had significantly higher odds of CKM progression than those in the lowest tertile, with ORs of 1.552 (95% CI: 1.223–1.971) and 3.755 (95% CI: 2.913–4.842), respectively (*P* for trend <0.001). These associations remained robust after additional adjustment for demographic and lifestyle factors in Model 2. After further adjustment for baseline CKM stage in Model 3, the ORs were 1.336 (95% CI: 1.048–1.704) for tertile 2 and 2.630 (95% CI: 2.004–3.451) for tertile 3, compared with tertile 1, with a significant trend across tertiles (*P* for trend <0.001). In additional analyses, participants in the highest-quartile ePWV group had significantly greater odds of progression to advanced CKM syndrome than those in the low ePWV group in the fully adjusted model (OR = 1.734; 95% CI: 1.415–2.125; *P* < 0.001). Quartile-based analysis also supported a graded association across higher ePWV categories. When ePWV was modeled as a continuous variable, each 1 m/s increase was associated with higher odds of CKM progression in the fully adjusted model (OR = 1.175; 95% CI: 1.090–1.267; *P* < 0.001).

**Table 2:** Association of ePWV with progression to advanced CKM syndrome in the CHARLS cohort.

ePWV	Model 1 OR (95% CI)	*P*-value	Model 2 OR (95% CI)	*P*-value	Model 3 OR (95% CI)	*P*-value
Cutoff						
Low	1 (reference)		1 (reference)		1 (reference)	
High	2.317 (1.911–2.808)	<0.001	2.329 (1.920–2.826)	<0.001	1.734 (1.415–2.125)	<0.001
Tertiles						
Tertile 1	1 (reference)		1 (reference)		1 (reference)	
Tertile 2	1.552 (1.223–1.971)	<0.001	1.562 (1.229–1.985)	<0.001	1.336 (1.048–1.704)	0.019
Tertile 3	3.755 (2.913–4.842)	<0.001	3.796 (2.940–4.902)	<0.001	2.630 (2.004–3.451)	<0.001
*P*-trend		<0.001		<0.001		<0.001
Quartiles						
Quartile 1	1 (reference)		1 (reference)		1 (reference)	
Quartile 2	1.493 (1.115–1.998)	0.007	1.515 (1.131–2.028)	0.005	1.341 (0.999–1.800)	0.051
Quartile 3	2.432 (1.836–3.221)	<0.001	2.446 (1.845–3.244)	<0.001	1.900 (1.423–2.537)	<0.001
Quartile 4	4.494 (3.302–6.117)	<0.001	4.552 (3.340–6.204)	<0.001	2.906 (2.091–4.041)	<0.001
*P*-trend		<0.001		<0.001		<0.001
Continuous						
Per 1 m/s increase	1.347 (1.260–1.440)	<0.001	1.354 (1.266–1.448)	<0.001	1.175 (1.090–1.267)	<0.001

Model 1 was adjusted for age and sex. Model 2 was further adjusted for education level, marital status, smoking status, and alcohol status. Model 3 was further adjusted for baseline CKM stage.

ePWV, estimated pulse wave velocity; CKM, cardiovascular–kidney–metabolic; CHARLS, China health and retirement longitudinal study; OR, odds ratio; CI, confidence interval.

### 3.3 Subgroup analyses

The positive association between higher ePWV and progression to advanced CKM syndrome was observed in all subgroups. Significant associations were found in both age strata, in both male and female participants, among alcohol drinkers and non-drinkers, and among participants with or without hypertension or diabetes. Significant interactions were observed by sex (*P* for interaction <0.001), alcohol status (*P* for interaction = 0.035), and history of hypertension (*P* for interaction = 0.033). The association appeared stronger in male than in female participants, stronger among alcohol drinkers than non-drinkers, and stronger among participants without hypertension than those with hypertension. No significant interactions were observed for age or history of diabetes (both *P* for interaction >0.05) (Table 3).

**Table 3:** Subgroup analyses of the association between ePWV and progression to advanced CKM syndrome in the CHARLS cohort.

Subgroup	T3 vs. T1 OR (95% CI)	*P*-value	*P*-interaction
Age			
<60 years	3.540 (2.618–4.789)	<0.001	0.882
≥60 years	3.546 (1.765–7.124)	<0.001	
Sex			
Male	3.164 (2.076–4.823)	<0.001	<0.001
Female	2.043 (1.430–2.919)	<0.001	
Alcohol status			
No	2.344 (1.689–3.253)	<0.001	0.035
Yes	3.300 (2.023–5.384)	<0.001	
History of hypertension			
No	2.447 (1.768–3.388)	<0.001	0.033
Yes	2.046 (1.173–3.570)	0.012	
History of diabetes			
No	2.931 (2.195–3.915)	<0.001	0.086
Yes	2.841 (1.073–7.523)	0.036	

ePWV, estimated pulse wave velocity; CKM, cardiovascular–kidney–metabolic; CHARLS, China health and retirement longitudinal study; OR, odds ratio; CI, confidence interval.

### 3.4 Sensitivity analyses

Among 7,801 eligible participants, 5,837 had available 2015 CKM follow-up data and 1,964 had missing 2015 CKM stage information or were lost to follow-up. Those with missing follow-up information differed from those with available data in several baseline demographic, lifestyle, and cardiometabolic characteristics, suggesting potential selection bias due to follow-up loss (Supplementary Table S3).

The inverse probability weighting sensitivity analysis showed results consistent with the primary complete-case analysis (Supplementary Table S4). In the fully adjusted weighted model, high ePWV remained significantly associated with increased odds of CKM progression (OR, 1.786; 95% CI, 1.444–2.210), with significant dose-response trends across tertiles and quartiles. Each 1 m/s increase in ePWV was also associated with increased odds of CKM progression (OR, 1.156; 95% CI, 1.067–1.253). These findings support the robustness of the main results after accounting for potential bias due to missing 2015 CKM follow-up data or loss to follow-up.

### 3.5 Longitudinal changes in ePWV and progression to advanced CKM syndrome

Greater increases in ePWV during follow-up were significantly associated with higher odds of progression to advanced CKM syndrome. In the fully adjusted model, each 1 m/s increase in change in ePWV was associated with 115.3% higher odds of CKM progression (OR = 2.153; 95% CI: 1.960–2.364; *P* < 0.001). Similarly, each 1-standard deviation increase in change in ePWV was associated with significantly higher odds of progression to advanced CKM syndrome (OR = 2.146; 95% CI: 1.955–2.355; *P* < 0.001). Consistent results were observed when change in ePWV was categorized into tertiles. Compared with participants in the lowest tertile, those in the highest tertile had markedly higher odds of progression to advanced CKM syndrome in the fully adjusted model (OR = 4.638; 95% CI: 3.688–5.833; *P* < 0.001), with a significant trend across tertiles (*P* for trend <0.001). Detailed estimates are presented in Table 4.

**Table 4:** Association of longitudinal changes in ePWV with progression to advanced CKM syndrome.

Exposure	Model 1 OR (95% CI)	*P*-value	Model 2 OR (95% CI)	*P*-value	Model 3 OR (95% CI)	*P*-value
Delta ePWV (per 1 m/s increase)	2.157 (1.967–2.366)	<0.001	2.196 (2.001–2.411)	<0.001	2.153 (1.960–2.364)	<0.001
Delta ePWV (per 1 SD increase)	2.150 (1.961–2.357)	<0.001	2.189 (1.995–2.402)	<0.001	2.146 (1.955–2.355)	<0.001
Delta ePWV tertiles						
Tertile 1	1 (reference)		1 (reference)		1 (reference)	
Tertile 2	1.832 (1.456–2.305)	<0.001	1.840 (1.461–2.317)	<0.001	1.829 (1.452–2.305)	<0.001
Tertile 3	4.630 (3.691–5.809)	<0.001	4.762 (3.792–5.981)	<0.001	4.638 (3.688–5.833)	<0.001
*P*-trend		<0.001		<0.001		<0.001

Model 1 was adjusted for age and sex. Model 2 was further adjusted for education level, marital status, smoking status, and alcohol status. Model 3 was further adjusted for baseline CKM stage.

ePWV, estimated pulse wave velocity; CKM, cardiovascular–kidney–metabolic; SD, standard deviation; OR, odds ratio; CI, confidence interval.

### 3.6 ePWV trajectory analysis

Among 5,404 participants with repeated ePWV measurements available, latent class mixed models with one to four classes were fitted to identify ePWV trajectories from 2011 to 2015. Although the four-class model had the lowest Bayesian information criterion, it generated small classes and showed less stable classification, including one class with a relatively low average posterior probability. Therefore, the two-class model was selected for parsimony, classification stability, and clinical interpretability (Supplementary Table S5).

**Figure 2:**
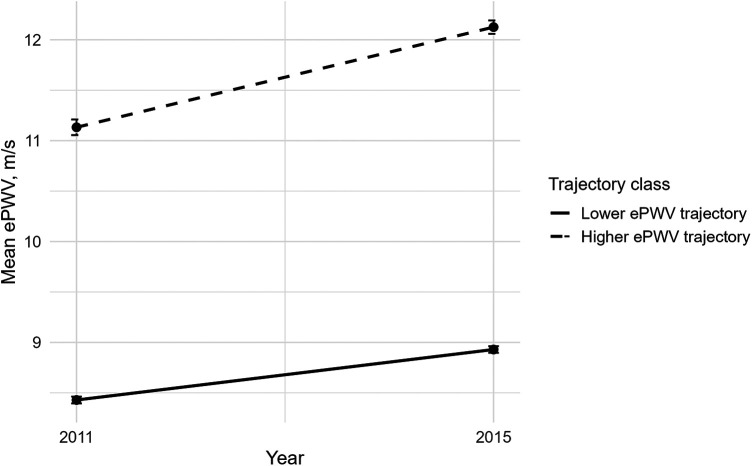
Trajectories of ePWV from 2011 to 2015. Points represent mean ePWV values at each time point, and error bars indicate 95% CIs. ePWV, estimated pulse wave velocity.

Participants in the lower ePWV trajectory group maintained relatively low ePWV levels with a modest increase over time, whereas those in the higher ePWV trajectory group had persistently elevated ePWV levels and showed a greater increase during follow-up (Figure 2). Compared with the lower ePWV trajectory group, the higher ePWV trajectory group had significantly greater odds of progression to advanced CKM syndrome after adjustment for age, sex, education, marital status, smoking status, alcohol status, and baseline CKM stage (OR = 4.696; 95% CI: 3.772–5.855; *P* < 0.001).

### 3.7 ROC analysis of baseline ePWV for predicting progression to advanced CKM syndrome

Baseline ePWV showed moderate discriminatory performance for predicting 4-year progression to advanced CKM syndrome (AUC, 0.722; 95% CI: 0.705–0.740; Supplementary Figure S2). At the ROC-derived optimal cutoff of 9.629, ePWV showed high negative predictive value, suggesting better ability to identify participants unlikely to progress (Supplementary Table S6). However, ePWV did not outperform models incorporating age and blood pressure before or after covariate adjustment, and DeLong tests showed significantly lower AUCs for ePWV than for these component-based models (Supplementary Table S7). Adding ePWV to the traditional CKM risk factor model resulted in only a small increase in AUC from 0.755 to 0.759 (*Δ*AUC = 0.0036; DeLong *P* = 0.020) (Supplementary Table S8). Although continuous net reclassification improvement and integrated discrimination improvement were statistically significant, categorical net reclassification improvement was not, indicating limited incremental predictive improvement beyond traditional CKM risk factors (Supplementary Table S8).

### 3.8 RCS analysis of the association between ePWV and progression to advanced CKM syndrome

**Figure 3:**
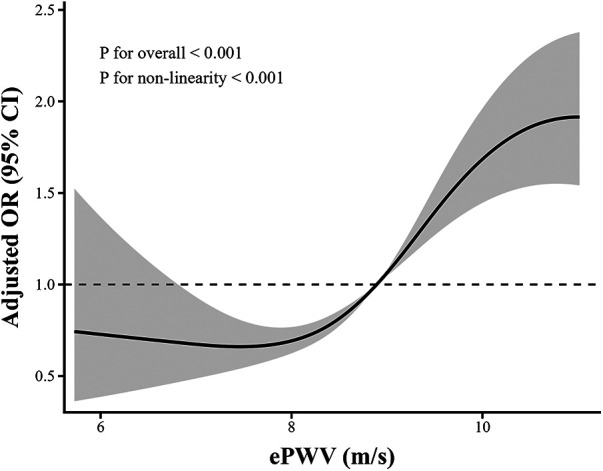
Dose-response relationship between baseline ePWV and progression to advanced CKM syndrome. The solid line represents the adjusted OR, and the shaded area indicates the 95% CI. ePWV, estimated pulse wave velocity; CKM, cardiovascular–kidney–metabolic; OR, odds ratio; CI, confidence interval.

RCS analysis showed a significant overall association and evidence of nonlinearity between baseline ePWV and progression to advanced CKM syndrome. The adjusted odds of CKM progression remained relatively stable at lower ePWV levels and increased more steeply above approximately 9 m/s. This nonlinear pattern remained significant in the fully adjusted model (*P* for overall <0.001; *P* for nonlinearity <0.001; Figure 3).

### 3.9 Supportive cross-sectional analysis in the hospital-based cohort

Among the 1,596 participants in the hospital-based cross-sectional cohort, 44 (2.8%) had advanced CKM syndrome (stages 3–4), whereas 1,552 (97.2%) had non-advanced CKM syndrome (stages 0–2). Compared with participants without advanced CKM syndrome, those with advanced CKM syndrome were older (65.2 ± 9.3 vs. 62.2 ± 10.8 years), more likely to be male (93.18% vs. 51.48%), and had higher SBP (144.2 ± 20.6 vs. 133.4 ± 18.8 mmHg) and DBP (81.2 ± 13.0 vs. 77.2 ± 11.3 mmHg). Characteristics of the hospital-based cross-sectional cohort are presented in Supplementary Table S9.

In this hospital-based cross-sectional cohort, higher ePWV was associated with greater odds of advanced CKM syndrome. When analyzed as a continuous variable, each 1 m/s increase in ePWV was significantly associated with higher odds of advanced CKM syndrome in both the unadjusted model (OR = 1.192; 95% CI: 1.054–1.349; *P* = 0.005) and the age- and sex-adjusted model (OR = 1.421; 95% CI: 1.047–1.929; *P* = 0.024). Tertile-based analysis also showed that participants in higher ePWV tertiles had greater odds of advanced CKM syndrome than those in the lowest tertile. After adjustment for age and sex, the ORs were 6.234 (95% CI: 1.961–19.818; *P* = 0.002) for tertile 2 and 6.144 (95% CI: 1.441–26.199; *P* = 0.014) for tertile 3, with a significant trend across tertiles (*P* for trend = 0.022) (Table 5). Additional analyses showed that the binary ePWV cutoff was positively but not significantly associated with advanced CKM syndrome after adjustment for age and sex, whereas quartile-based analyses showed generally positive graded associations; these results are presented in (Supplementary Table S10). The ePWV-based model showed modest cross-sectional discrimination for advanced CKM syndrome, with an AUC of 0.649 (95% CI: 0.584–0.715) (Supplementary Figure S3).

**Table 5:** Main supportive cross-sectional analysis of ePWV and advanced CKM syndrome in the hospital-based cohort.

Exposure	Model 1 OR (95% CI)	*P*-value	Model 2 OR (95% CI)	*P*-value
Tertiles				
Tertile 1	Reference		Reference	
Tertile 2	5.425 (1.849–15.912)	0.002	6.234 (1.961–19.818)	0.002
Tertile 3	4.889 (1.652–14.468)	0.004	6.144 (1.441–26.199)	0.014
*P*-trend		0.006		0.022
Continuous				
ePWV (per 1 m/s increase)	1.192 (1.054–1.349)	0.005	1.421 (1.047–1.929)	0.024

Model 1 was unadjusted. Model 2 was adjusted for age and sex.

ePWV, estimated pulse wave velocity; CKM, cardiovascular–kidney–metabolic; OR, odds ratio; CI, confidence interval.

## 4 Discussion

In this population-based longitudinal analysis of the CHARLS cohort, higher ePWV was associated with increased odds of progression to advanced CKM syndrome. This finding was further supported by cross-sectional evidence from an independent hospital-based cross-sectional cohort, in which higher ePWV was consistently associated with the presence of advanced CKM syndrome when analyzed both continuously and according to tertiles. In addition, both longitudinal increases in ePWV and a persistently high ePWV trajectory were associated with greater odds of CKM progression. These findings suggest that worsening or sustained arterial stiffness may reflect the transition toward advanced CKM stages and highlight the potential value of ePWV as a practical marker for risk stratification in CKM syndrome.

Previous studies have linked higher ePWV to adverse outcomes across cardiovascular, metabolic, renal, and hepatic conditions (9–13, 19, 20). More recent studies have also associated elevated ePWV with mortality among patients with CKD and atherosclerotic heart disease (21), CVD risk in individuals with CKM syndrome (14), and incident stroke among those with CKM stages 0–3 (16). However, most prior studies have focused on mortality, incident CVD, stroke, or individual cardiometabolic and renal outcomes. Evidence regarding progression across integrated CKM stages remains limited. Our study extends this evidence by applying an operational CKM staging framework based on the AHA CKM syndrome classification, showing that both higher baseline ePWV and longitudinal increases in ePWV were associated with progression from early to advanced CKM syndrome. Taken together, these findings suggest that ePWV may serve as an accessible marker associated with worsening CKM health, but it should be interpreted as a surrogate indicator of vascular aging rather than a direct assessment of arterial stiffness or atherosclerotic burden.

Subgroup analyses showed generally consistent associations between ePWV and CKM progression across major clinical strata. Notably, ePWV was more strongly associated with CKM progression in men, a pattern consistent with previous evidence of sex-specific vascular aging (22). This finding may reflect sex-related differences in vascular aging and cardiometabolic risk accumulation. Men may have a higher burden of adverse metabolic and hemodynamic factors, making elevated ePWV a more sensitive marker of accumulated vascular risk (23, 24). In women, sex hormone-related vascular protection, particularly before menopause, may partly attenuate vascular stiffening and its cardiometabolic consequences (23, 24). However, this subgroup finding should be interpreted cautiously because the analysis was exploratory and information on menopausal status, sex hormones, and detailed lifestyle factors was unavailable. Further studies are needed to determine whether sex-specific ePWV thresholds or prediction models can improve CKM risk stratification. The association was also stronger among alcohol drinkers than among non-drinkers. This finding may reflect the potential contribution of alcohol-related vascular injury, elevated blood pressure, inflammation, oxidative stress, or metabolic disturbances to CKM progression (25). However, it should be interpreted cautiously because alcohol status was self-reported, detailed information on the amount and pattern of alcohol consumption was unavailable, and subgroup analyses may be affected by residual confounding and multiple testing. In addition, the association appeared stronger among participants without hypertension than among those with hypertension, suggesting that ePWV may reflect early vascular aging and cardiometabolic risk before the development of clinically recognized hypertension (26). Among participants with diabetes, the association remained statistically significant, but the CI was relatively wide, indicating limited statistical precision, possibly due to the smaller subgroup size and fewer outcome events. The high baseline vascular and cardiometabolic risk burden among participants with diabetes may also make the additional contribution of ePWV more difficult to distinguish, possibly reflecting a high-risk background or partial ceiling effect (27). Importantly, the interaction by diabetes status did not reach conventional statistical significance, indicating no clear evidence of diabetes-specific effect modification. Overall, these subgroup findings should be interpreted cautiously and require further validation.

Longitudinal analyses further suggest that arterial stiffness is not merely a static risk marker but a dynamic process linked to CKM progression, with increasing ePWV potentially reflecting ongoing vascular aging, cumulative hemodynamic burden, endothelial dysfunction, and worsening metabolic–renal–cardiovascular interactions (17, 28, 29). The trajectory analysis further supports this interpretation. Participants with persistently high ePWV levels and greater increases during follow-up may represent a subgroup with sustained or progressive vascular stiffening, rather than risk captured by a single baseline measurement alone. This pattern is clinically and public-health relevant because sustained arterial stiffening may accompany or signal the transition to advanced CKM stages and may help identify individuals whose risk is increasing over time. Repeated assessment of ePWV may therefore provide additional information beyond baseline measurement and may be useful for monitoring vascular health and refining early CKM risk stratification.

The hospital-based analysis provided supportive evidence from an external cross-sectional setting for the association between higher ePWV and prevalent advanced CKM syndrome, but this evidence should be interpreted cautiously. The limited number of participants with advanced CKM syndrome may have resulted in sparse data in some ePWV categories, reduced statistical power, and wider CIs. The attenuation of the cutoff-based association after adjustment for age and sex may also reflect information loss from dichotomizing a continuous variable and the strong dependence of ePWV on age. In addition, because the hospital-based cohort was derived from routine health examination records, detailed covariate information was limited, and the supportive cross-sectional analysis included only age and sex as covariates. Thus, its adjustment strategy was less comprehensive than that used in the CHARLS models. Accordingly, residual confounding by insufficiently captured factors, including smoking and drinking status, socioeconomic characteristics, antihypertensive, glucose-lowering, or lipid-lowering medication use, pre-existing comorbidities, and differences in health-seeking behavior, cannot be excluded. For example, antihypertensive treatment may affect blood pressure and thereby influence calculated ePWV, while medication use and comorbid disease burden may also be associated with CKM stage. Accordingly, the hospital-based cohort should be viewed as providing supportive cross-sectional evidence rather than definitive external validation of longitudinal CKM progression. Larger external cohorts with prospective follow-up and more complete information on lifestyle factors, medication use, comorbidities, socioeconomic characteristics, and longitudinal CKM outcomes are needed to confirm these findings.

Several mechanisms may explain the association between higher ePWV and progression to advanced CKM stages. First, increased arterial stiffness elevates pulse pressure and left ventricular afterload, which may contribute to cardiovascular damage and atherosclerotic progression (6, 29). Second, loss of arterial elasticity may increase pulsatile pressure transmission into the microcirculation, particularly in the kidneys, thereby contributing to renal dysfunction (5, 12). Third, metabolic disorders, including insulin resistance, obesity, dyslipidemia, and chronic low-grade inflammation, may exacerbate endothelial injury and further increase arterial stiffness (30). These processes are interconnected. Vascular stiffening may accelerate renal and cardiovascular injury, while metabolic and renal abnormalities may in turn aggravate vascular dysfunction (31). Collectively, elevated ePWV may reflect the cumulative burden of vascular, metabolic, renal, and cardiovascular deterioration during CKM progression.

Because ePWV is calculated from age and MBP, its association with progression to advanced CKM syndrome should be interpreted as reflecting the combined vascular risk burden related to aging and elevated blood pressure. Supplementary analyses showed that ePWV had predictive performance comparable to, but not superior to, models directly incorporating age and blood pressure; therefore, it should not be interpreted as providing predictive information fully independent of its component variables. Furthermore, because ePWV was calculated using blood pressure measured at the time of examination, ePWV values among participants receiving antihypertensive therapy reflect treated rather than untreated blood pressure. Treatment-related reductions in blood pressure may result in a lower calculated ePWV than would have been observed in the untreated state, although this difference may partly reflect a genuine reduction in hemodynamic load and a potential reduction in functional arterial stiffness. Nevertheless, current treated blood pressure may not fully capture the cumulative vascular damage associated with long-standing or previously uncontrolled hypertension. Consequently, treated individuals may retain substantial arterial stiffness and cardiovascular risk despite having a lower calculated ePWV. In addition, participants receiving antihypertensive treatment may have had more severe or longer-standing hypertension, potentially introducing confounding by indication. Although hypertension status and baseline CKM stage were considered in the analyses, these adjustments may not fully account for treatment-related confounding. Detailed information on antihypertensive medication classes, treatment duration, adherence, and pretreatment blood pressure was unavailable; therefore, treatment-related exposure misclassification and residual confounding cannot be excluded. Moreover, ePWV is not a direct measure of arterial stiffness or atherosclerotic progression. Carotid–femoral pulse wave velocity remains the reference standard for assessing arterial stiffness, whereas coronary computed tomography angiography, multislice computed tomography, and carotid ultrasonography provide more direct information on atherosclerotic plaque burden and vascular structural changes (32, 33). Nevertheless, ePWV may have practical value as a simple and scalable vascular-aging index for preliminary CKM risk stratification, especially when direct arterial stiffness measurement or vascular imaging is unavailable. Because no universally accepted ePWV threshold for predicting progression to advanced CKM syndrome exists, the ROC-derived cutoff and related sensitivity, specificity, positive predictive value, and negative predictive value should be interpreted as exploratory. In practice, elevated or increasing ePWV may help identify individuals who warrant closer follow-up and more comprehensive management of blood pressure, metabolic abnormalities, renal dysfunction, and cardiovascular risk factors. Thus, ePWV should be considered a complementary marker rather than a substitute for imaging-based vascular assessment, and our findings should be interpreted as supporting its predictive value for relatively short-term progression to advanced CKM syndrome over 4 years. Further prospective studies with longer follow-up are needed to validate its prognostic value and determine whether repeated ePWV assessment can improve CKM risk monitoring.

This study has several limitations. First, ePWV was calculated from age and blood pressure rather than measured directly as arterial stiffness. Because direct carotid–femoral pulse wave velocity measurement and vascular imaging data were unavailable, we could not directly evaluate arterial stiffness, atherosclerotic plaque burden, or plaque phenotype. In addition, overlap in components related to aging and blood pressure cannot be fully excluded because both ePWV and several CKM staging criteria are associated with age and blood pressure. Second, exclusion of participants with incomplete baseline CKM staging information or missing 2015 follow-up data may have introduced selection bias, although the inverse probability weighting sensitivity analysis supported the robustness of the main findings. Third, the observational design precludes causal inference, and residual confounding remains possible because information on medication use, physical activity, diet, inflammatory markers, and disease duration was limited. Fourth, the 4-year follow-up period was relatively short for CKM syndrome; therefore, our findings should be interpreted as reflecting short-term progression to advanced CKM syndrome rather than long-term CKM-related outcomes. Fifth, the hospital-based cohort was cross-sectional and adjusted only for age and sex; therefore, it should be interpreted as supportive evidence for prevalent advanced CKM syndrome rather than external validation of longitudinal CKM progression. Finally, the study population was restricted to adults aged 45 years or older, which may limit generalizability to younger populations.

## 5 Conclusion

This study showed that higher baseline ePWV, larger longitudinal increases in ePWV, and a persistently higher ePWV trajectory were associated with 4-year progression to advanced CKM syndrome, with supportive evidence from an independent hospital-based cross-sectional cohort. Because ePWV can be easily calculated from routinely measured age and blood pressure, it may serve as a simple and low-cost marker for identifying individuals at higher risk of CKM progression in routine health examinations, primary care, and outpatient settings. Individuals with elevated or increasing ePWV may benefit from closer follow-up and earlier management of blood pressure, metabolic, weight-related, and kidney-related risk factors. However, because this observational study cannot determine whether lowering ePWV or using ePWV-guided risk stratification can prevent CKM progression, further studies with longer follow-up and interventional designs are needed to clarify the long-term prognostic value and clinical utility of ePWV in CKM prevention.

## Data Availability

The raw data supporting the conclusions of this article will be made available by the authors, without undue reservation.
