# Supplementary material for: Estimated pulse wave velocity and progression to advanced cardiovascular–kidney–metabolic syndrome: a population-based longitudinal study with supportive external cross-sectional evidence
Source: Front Cardiovasc Med. 2026 Jul 8;13:1875961. doi: 10.3389/fcvm.2026.1875961 (PMC13388791; doi:10.3389/fcvm.2026.1875961)
Supplement: Supplementary file 1 [file Datasheet1.pdf]

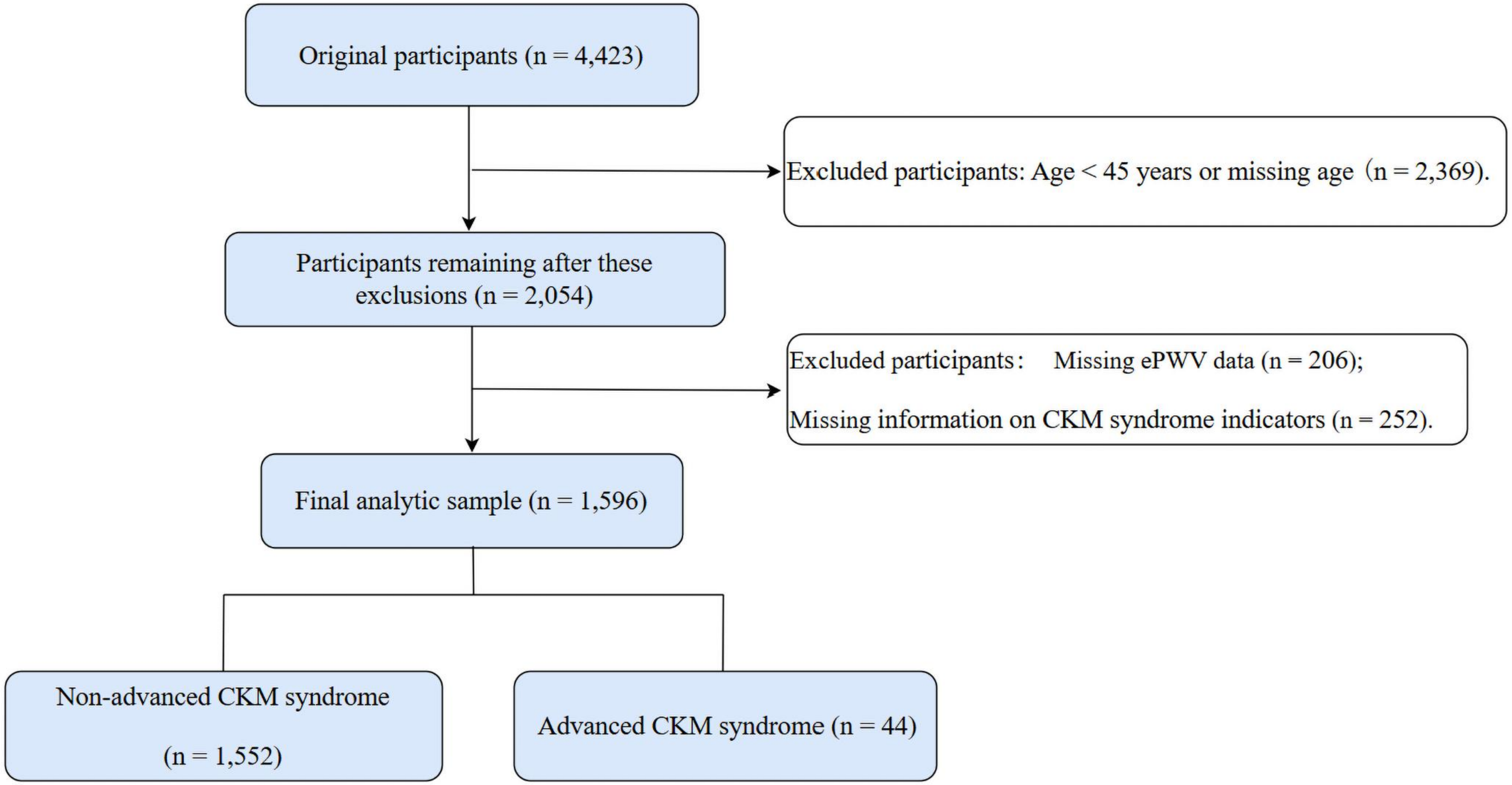

Figure S1. Flowchart of participant selection for the hospital-based cross-sectional cohort.

Abbreviations: ePWV, estimated pulse wave velocity; CKM, cardiovascular–kidney–metabolic.

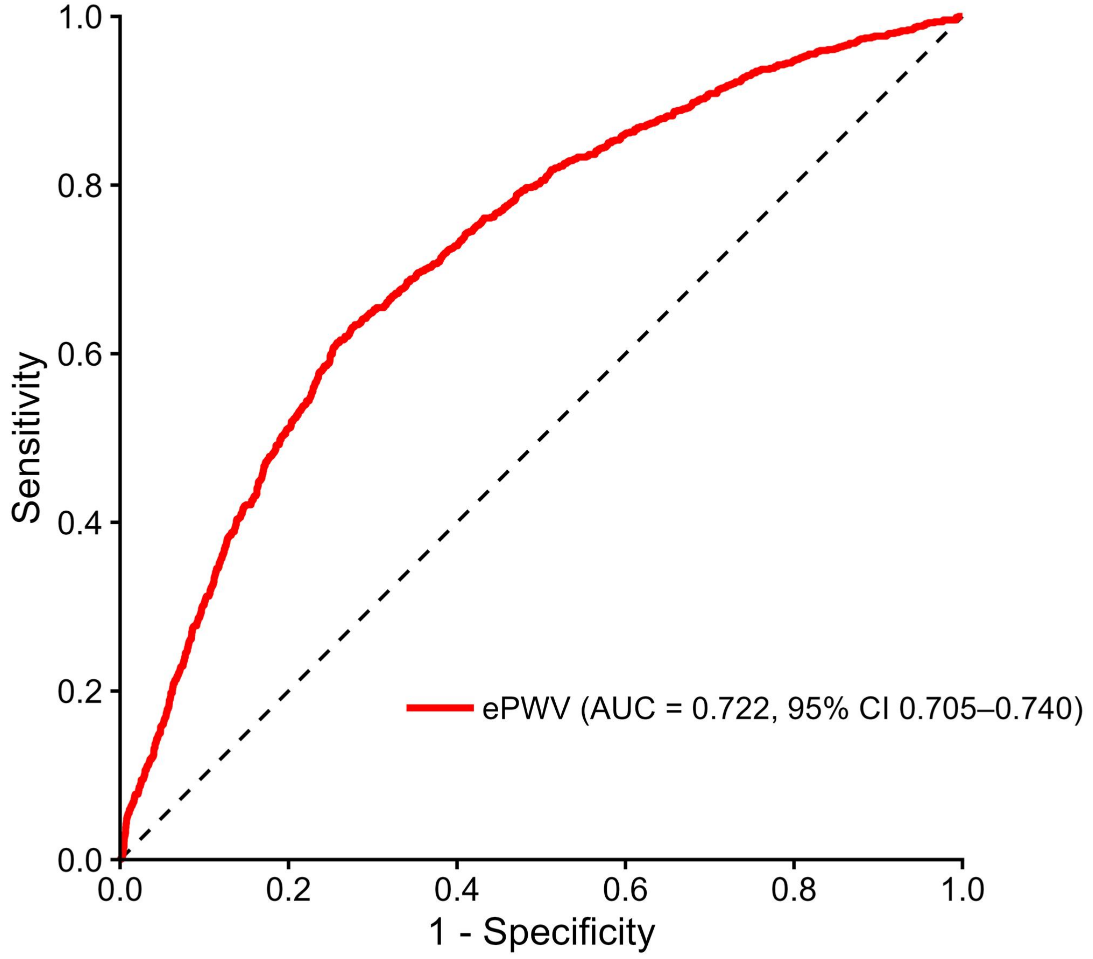

Figure S2. ROC curve of baseline ePWV for predicting progression to advanced CKM syndrome in the CHARLS cohort.

Abbreviations: ROC, receiver operating characteristic; ePWV, estimated pulse wave velocity; CKM, cardiovascular–kidney–metabolic; CHARLS, China Health and Retirement Longitudinal Study; AUC, area under the curve; CI, confidence interval.

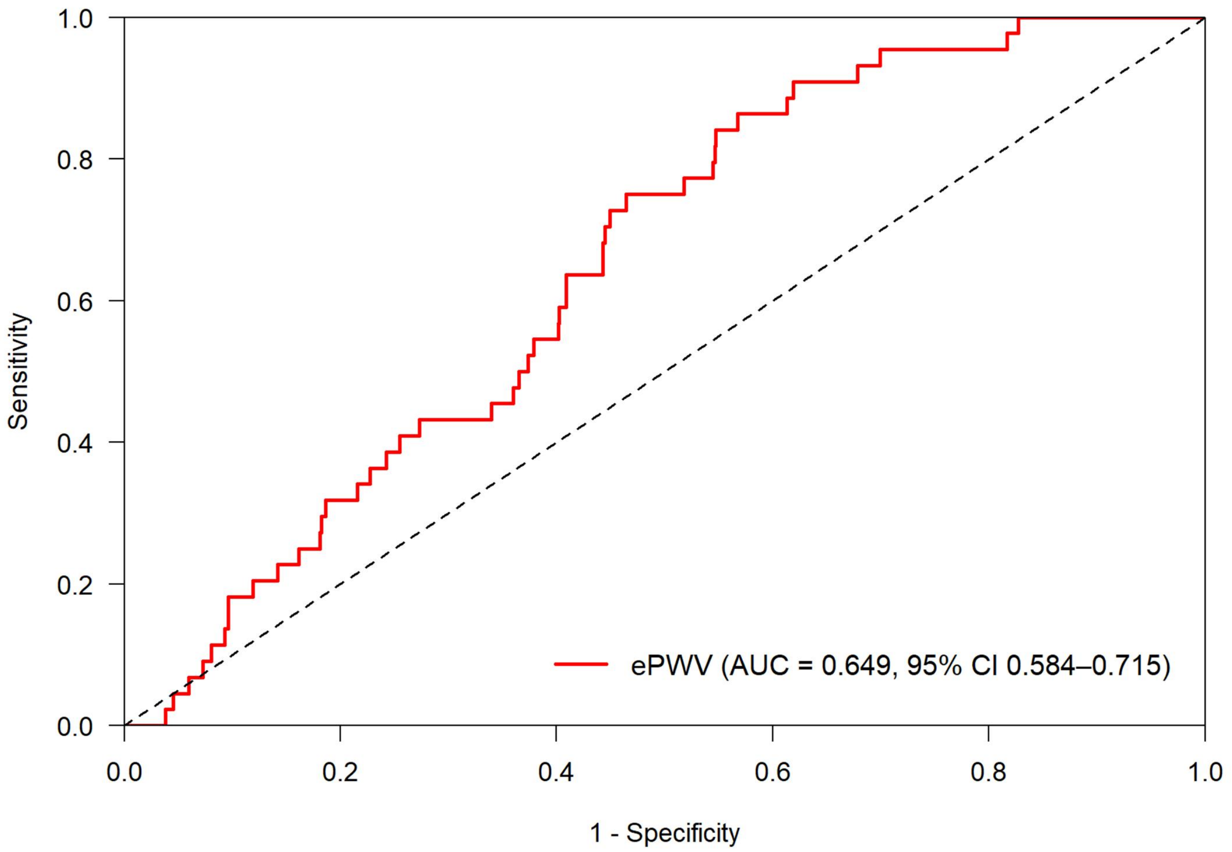

Figure S3. ROC curve of ePWV for identifying advanced CKM syndrome in the hospital-based cross-sectional cohort.

Abbreviations: ROC, receiver operating characteristic; ePWV, estimated pulse wave velocity; CKM, cardiovascular−kidney−metabolic; AUC, area under the curve; CI, confidence interval.

Table S1. Definition criteria for CKM syndrome stages.

CKM syndrome stages	Definition
**Stage 0**	Normal BMI (< 23 kg/m^2);<br>Normal waist circumference (< 80 cm in women or < 90 cm in men);<br>Normoglycemia;<br>Normotension;<br>Normal lipid status;<br>No evidence of CKD;<br>No evidence of CVD;<br>Must meet all listed criteria.
**Stage 1**	Elevated BMI (≥ 23 kg/m^2);<br>Elevated waist circumference (≥ 80 cm in women or ≥ 90 cm in men);<br>Prediabetes (fasting blood glucose between 100 mg/dL and 125 mg/dL or glycated hemoglobin between 5.7% and 6.4%).<br>Meeting any one of the listed criteria is sufficient.
**Stage 2**	Elevated triglycerides (≥ 135 mg/dL);<br>Hypertension (blood pressure ≥ 140/90 mmHg, self-reported history of hypertension, or current antihypertensive treatment);<br>Diabetes (fasting blood glucose ≥ 126 mg/dL, glycated hemoglobin ≥ 6.5%, self-reported history of diabetes, or current hypoglycemic treatment); Metabolic syndrome (≥ 3 of the following: elevated waist circumference, HDL-C < 40 mg/dL (men) /< 50 mg/dL (women), triglycerides ≥ 150 mg/dL, blood pressure ≥ 130/80 mmHg and/or current antihypertensive treatment, fasting glucose ≥ 100 mg/dL);<br>Moderate-risk CKD[1].<br>Meeting any one of the listed criteria is sufficient.
**Stage 3**	Very-high-risk CKD[1]; High predicted 10-year CVD risk[2].<br>Meeting any one of the listed criteria is sufficient.
**Stage 4**	Self-reported established CVD (coronary heart disease, angina, heart attack, heart failure, and stroke).

1 CKD stages were identified based on estimated glomerular filtration rate. Moderate-risk CKD was defined as an estimated glomerular filtration rate of 30–59 mL/min/1.73 m², and very-high-risk CKD was defined as an estimated glomerular filtration rate < 30 mL/min/1.73 m².

2 The 10-year CVD risk was estimated using the Framingham General Cardiovascular Risk Score equations and high predicted 10-year CVD risk was defined as a predicted risk of ≥20%.

Abbreviations: CKM, cardiovascular–kidney–metabolic; BMI, body mass index; CKD, chronic kidney disease; CVD, cardiovascular disease; HDL-C, high-density lipoprotein cholesterol.

Table S2. Baseline characteristics of participants excluded because of missing baseline CKM staging information compared with the final analytic sample.

Characteristic	Excluded because baseline CKM stage was missing (N=3,070)	Included in final analytic sample (N=5,810)	SMD	P-value
Age, years	57.5 ± 10	56.9 ± 8.4	0.059	0.007
Sex, n (%)			0.461	< 0.001
Male	1,780 (58)	2,033 (35)		
Female	1,290 (42)	3,777 (65)		
Education, n (%)			0.204	< 0.001
Primary school or below	1,863 (61)	3,940 (68)		
Middle school	667 (22)	1,281 (22)		
High school or above	526 (17)	589 (10)		
Missing	14 (0.5)	0 (0.0)		
Marital status, n (%)			0.087	< 0.001
Married	2,726 (89)	5,191 (89)		
Previously married	296 (9.6)	591 (10)		
Never married	40 (1.3)	28 (0.5)		
Missing	8 (0.3)	0 (0.0)		
Smoking status, n (%)			0.561	< 0.001
Non-smoker	1,738 (57)	4,653 (80)		
Current smoker	884 (29)	1,157 (20)		
Missing	448 (15)	0 (0.0)		
Alcohol status, n (%)			0.249	< 0.001
Non-drinker	1,805 (59)	4,101 (71)		
Drinker	1,171 (38)	1,709 (29)		
Missing	94 (3.1)	0 (0.0)		
SBP, mmHg	116.9 ± 12.1	128.5 ± 19.7	0.71	< 0.001
DBP, mmHg	69.3 ± 9	75.9 ± 11.9	0.628	< 0.001
MBP, mmHg	88.4 ± 9.4	97 ± 13.9	0.724	< 0.001
BMI, kg/m²	20.2 ± 1.8	24 ± 3.7	1.308	< 0.001
Waist circumference, cm	76 ± 5.6	86.1 ± 9.6	1.279	< 0.001
Fasting glucose, mmol/L	5.1 ± 0.4	5.8 ± 1.4	0.719	< 0.001
HbA1c, %	5 ± 0.4	5.2 ± 0.7	0.422	< 0.001
Triglycerides, mg/dL	83.8 ± 25	123.7 ± 78.8	0.682	< 0.001
HDL-C, mg/dL	54.2 ± 15.1	52.9 ± 15.1	0.082	0.049
Total cholesterol, mg/dL	176.9 ± 33.7	191.8 ± 36.6	0.423	< 0.001
eGFR, mL/min/1.73 m²	96.7 ± 12.8	95.2 ± 13.2	0.113	0.008
ePWV, m/s	8.6 ± 1.6	9.1 ± 1.7	0.315	< 0.001
History of hypertension, n (%)			0.657	< 0.001
No	2,968 (97)	4,640 (80)		
Yes	0 (0.0)	1,132 (19)		
Missing	102 (3.3)	38 (0.7)		
History of diabetes, n (%)			0.3	< 0.001
No	2,960 (96)	5,506 (95)		
Yes	0 (0.0)	255 (4.4)		
Missing	110 (3.6)	49 (0.8)		
History of heart disease, n (%)				
No	2,968 (97)	5,768 (99)		
Missing	102 (3.3)	42 (0.7)		
History of stroke, n (%)				
No	2,980 (97)	5,796 (99.8)		
Missing	90 (2.9)	14 (0.2)		

P-values and SMDs were not estimated for variables with only one non-missing category.

Abbreviations: CKM, cardiovascular–kidney–metabolic; SMD, standardized mean difference; SBP, systolic blood pressure; DBP, diastolic blood pressure; MBP, mean blood pressure; BMI, body mass index; HbA1c, glycated hemoglobin; HDL-C, high-density lipoprotein cholesterol; eGFR, estimated glomerular filtration rate; ePWV, estimated pulse wave velocity.

Table S3. Baseline characteristics of participants with and without 2015 CKM follow-up information.

Characteristic	Had 2015 CKM follow-up information (N=5,837)	Missing 2015 CKM stage or lost to follow-up (N=1,964)	SMD	P-value
Age, years	56.9 ± 8.4	58.1 ± 10.3	0.125	< 0.001
Sex, n (%)			0.197	< 0.001
Male	2,043 (35)	877 (45)		
Female	3,787 (65)	1,086 (55)		
Missing	7 (0.1)	1 (0.1)		
Education, n (%)			0.138	< 0.001
Primary school or below	3,963 (68)	1,266 (64)		
Middle school	1,283 (22)	408 (21)		
High school or above	591 (10)	288 (15)		
Missing	0 (0.0)	2 (0.1)		
Marital status, n (%)			0.108	< 0.001
Married	5,214 (89)	1,684 (86)		
Previously married	594 (10)	263 (13)		
Never married	29 (0.5)	17 (0.9)		
Smoking status, n (%)			0.149	< 0.001
Non-smoker	4,658 (80)	1,436 (73)		
Current smoker	1,159 (20)	510 (26)		
Missing	20 (0.3)	18 (0.9)		
Alcohol status, n (%)			0.046	0.084
Non-drinker	4,114 (70)	1,343 (68)		
Drinker	1,717 (29)	619 (32)		
Missing	6 (0.1)	2 (0.1)		
SBP, mmHg	128.5 ± 19.7	128.5 ± 20.7	0.000	0.985
DBP, mmHg	75.9 ± 11.9	75.5 ± 12.3	0.038	0.148
MBP, mmHg	97.0 ± 13.9	96.7 ± 14.3	0.020	0.450
BMI, kg/m²	24.0 ± 3.8	23.1 ± 3.8	0.251	< 0.001
Waist circumference, cm	86.1 ± 9.6	84.0 ± 9.8	0.219	< 0.001
Fasting glucose, mmol/L	5.8 ± 1.4	5.7 ± 1.4	0.077	0.024
HbA1c, %	5.2 ± 0.7	5.1 ± 0.7	0.067	0.040
Triglycerides, mg/dL	123.7 ± 78.8	112.5 ± 70.2	0.146	< 0.001
HDL-C, mg/dL	52.9 ± 15.0	55.0 ± 14.9	0.140	< 0.001
Total cholesterol, mg/dL	191.7 ± 36.6	190.2 ± 37.7	0.041	0.209
eGFR, mL/min/1.73 m²	95.2 ± 13.2	94.0 ± 14.2	0.086	0.009
ePWV, m/s	9.1 ± 1.7	9.3 ± 2.1	0.112	< 0.001
History of hypertension, n (%)			0.189	< 0.001
No	4,658 (80)	1,703 (87)		
Yes	1,136 (19)	247 (13)		
Missing	43 (0.7)	14 (0.7)		
History of diabetes, n (%)			0.089	0.002
No	5,526 (95)	1,887 (96)		
Yes	257 (4.4)	54 (2.7)		
Missing	54 (0.9)	23 (1.2)		
History of heart disease, n (%)				
No	5,790 (99)	1,951 (99)		
Missing	47 (0.8)	13 (0.7)		
History of stroke, n (%)				
No	5,818 (99.7)	1,961 (99.8)		
Missing	19 (0.3)	3 (0.2)		

P-values and SMDs were not estimated for variables with only one non-missing category.

Abbreviations: CKM, cardiovascular–kidney–metabolic; SMD, standardized mean difference; SBP, systolic blood pressure; DBP, diastolic blood pressure; MBP, mean blood pressure; BMI, body mass index; HbA1c, glycated hemoglobin; HDL-C, high-density lipoprotein cholesterol; eGFR, estimated glomerular filtration rate; ePWV, estimated pulse wave velocity.

Table S4. IPW sensitivity analysis for the association between baseline ePWV and progression to advanced CKM syndrome.

ePWV	Model 1 OR (95% CI)	P-value	Model 2 OR (95% CI)	P-value	Model 3 OR (95% CI)	P-value
Cutoff						
Low	Reference		Reference		Reference	
High	2.396 (1.958–2.933)	< 0.001	2.401 (1.959–2.943)	< 0.001	1.786 (1.444–2.210)	< 0.001
Tertiles						
Tertile 1	Reference		Reference		Reference	
Tertile 2	1.587 (1.246–2.022)	< 0.001	1.606 (1.260–2.046)	< 0.001	1.380 (1.081–1.761)	0.010
Tertile 3	3.885 (2.988–5.052)	< 0.001	3.930 (3.016–5.120)	< 0.001	2.744 (2.067–3.643)	< 0.001
P-trend		< 0.001		< 0.001		< 0.001
Quartiles						
Quartile 1	Reference		Reference		Reference	
Quartile 2	1.456 (1.082–1.958)	0.013	1.484 (1.103–1.997)	0.009	1.319 (0.978–1.780)	0.070
Quartile 3	2.445 (1.837–3.254)	< 0.001	2.471 (1.855–3.292)	< 0.001	1.937 (1.445–2.598)	< 0.001
Quartile 4	4.607 (3.350–6.337)	< 0.001	4.670 (3.390–6.433)	< 0.001	3.016 (2.142–4.249)	< 0.001
P-trend		< 0.001		< 0.001		< 0.001
Continuous						
Per 1 m/s increase	1.328 (1.238–1.425)	< 0.001	1.337 (1.245–1.436)	< 0.001	1.156 (1.067–1.253)	< 0.001

Model 1 was adjusted for age and sex. Model 2 was further adjusted for education level, marital status, smoking status, and alcohol status. Model 3 was further adjusted for baseline CKM stage.

Abbreviations: IPW, inverse probability weighting, ePWV, estimated pulse wave velocity; CKM, cardiovascular–kidney–metabolic; OR, odds ratio; CI, confidence interval.

Table S5. Model Fit and Classification Diagnostics for Latent Class Mixed Models of ePWV Trajectories

Model	Converged	Log-likelihood	AIC	BIC	ΔBIC	Class	n	Proportion, %	Average posterior probability
1-class model	Yes	-17970.04	35948.08	35974.46	760.21	1	5404	100	1
2-class model	Yes	-17658.33	35332.66	35385.42	171.17	1	4091	75.7	0.832
2-class model	Yes	-17658.33	35332.66	35385.42	171.17	2	1313	24.3	0.815
3-class model	Yes	-17598.85	35221.7	35300.84	86.59	1	4049	74.93	0.839
3-class model	Yes	-17598.85	35221.7	35300.84	86.59	2	1274	23.58	0.798
3-class model	Yes	-17598.85	35221.7	35300.84	86.59	3	81	1.5	0.731
4-class model	Yes	-17538.36	35108.73	35214.25	0	1	942	17.43	0.741
4-class model	Yes	-17538.36	35108.73	35214.25	0	2	322	5.96	0.678
4-class model	Yes	-17538.36	35108.73	35214.25	0	3	4049	74.93	0.829
4-class model	Yes	-17538.36	35108.73	35214.25	0	4	91	1.68	0.76

Abbreviations: ePWV, estimated pulse wave velocity; AIC, Akaike information criterion; BIC, Bayesian information criterion; **Δ**BIC, difference in BIC relative to the model with the lowest BIC; n, number of participants.

Table S6. Exploratory diagnostic performance of ePWV for predicting 4-year progression to advanced CKM syndrome

Predictor	N	Events, n (%)	Cutoff	AUC (95% CI)	Sensitivity	Specificity	PPV	NPV	Accuracy	Youden
ePWV	5810	941 (16.2%)	9.629	0.722 (0.705–0.740)	63.10%	72.5%	30.7%	91%	70.90%	0.356

Abbreviations: ePWV, estimated pulse wave velocity; CKM, cardiovascular–kidney–metabolic; N, number of participants; n, number of events; AUC, area under the curve; CI, confidence interval; PPV, positive predictive value; NPV, negative predictive value.

Table S7. Predictive performance of ePWV versus age- and blood pressure-based models for CKM progression

Model type	Model	AUC (95% CI)	ΔAUC vs. ePWV	DeLong P-value
Unadjusted model	ePWV	0.722 (0.705–0.740)	Reference	Reference
Unadjusted model	Age + MBP	0.733 (0.715–0.750)	0.010	<0.001
Unadjusted model	Age + SBP + DBP	0.733 (0.716–0.750)	0.010	<0.001
Unadjusted model	Formula-related raw components	0.733 (0.716–0.750)	0.011	<0.001
Covariate-adjusted model	ePWV + covariates	0.741 (0.724–0.758)	Reference	Reference
Covariate-adjusted model	Age + MBP + covariates	0.755 (0.738–0.772)	0.014	<0.001
Covariate-adjusted model	Age + SBP + DBP + covariates	0.755 (0.738–0.772)	0.014	<0.001
Covariate-adjusted model	Formula-related raw components + covariates	0.761 (0.744–0.778)	0.020	<0.001

Abbreviations: ePWV, estimated pulse wave velocity; CKM, cardiovascular–kidney–metabolic; AUC, area under the curve; CI, confidence interval; MBP, mean blood pressure; SBP, systolic blood pressure; DBP, diastolic blood pressure.

Table S8. Incremental predictive value of ePWV beyond traditional CKM risk factors

Metric	Estimate	95% CI	P-value
AUC of traditional model	0.755	0.738-0.772	
AUC of traditional model + ePWV	0.759	0.742-0.776	
Delta AUC	0.0036	0.001-0.007	0.020
Continuous NRI	0.335	0.260-0.417	<0.001
Categorical NRI	-0.003	-0.020-0.03	0.774
IDI	0.0042	0.001-0.009	0.004

Abbreviations: ePWV, estimated pulse wave velocity; CKM, cardiovascular–kidney–metabolic; CI, confidence interval; AUC, area under the curve; NRI, net reclassification improvement; IDI, integrated discrimination improvement.

Table S9. Baseline characteristics of participants in the hospital-based cross-sectional cohort according to advanced CKM syndrome status

Characteristic	Overall (N=1,596)	Stage 0–2 (N=1,552)	Stage 3–4 (N=44)	P-value
Age, years	62.3 ± 10.8	62.2 ± 10.8	65.2 ± 9.3	0.004
Sex, n (%)				< 0.001
Male	840 (52.63)	799 (51.48)	41 (93.18)	
Female	756 (47.37)	753 (48.52)	3 (6.82)	
SBP, mmHg	133.7 ± 18.9	133.4 ± 18.8	144.2 ± 20.6	0.001
DBP, mmHg	77.3 ± 11.3	77.2 ± 11.3	81.2 ± 13	0.048

Abbreviations: CKM, cardiovascular ‑ kidney ‑ metabolic; SBP, systolic blood pressure; DBP, diastolic blood pressure.

Table S10. Supplementary cross-sectional analysis of ePWV and advanced CKM syndrome in the hospital-based cohort

Exposure	Model 1 OR (95% CI)	P-value	Model 2 OR (95% CI)	P-value
ePWV cutoff				
Low	Reference		Reference	
High	1.928 (1.040–3.577)	0.037	1.611 (0.626–4.144)	0.323
Quartiles				
Quartile 1	Reference		Reference	
Quartile 2	4.581 (0.983–21.337)	0.053	6.027 (1.254–28.961)	0.025
Quartile 3	8.292 (1.894–36.308)	0.005	17.223 (3.339–88.831)	<0.001
Quartile 4	8.834 (2.027–38.493)	0.004	28.892 (4.358–191.571)	<0.001
P-trend		<0.001		<0.001

Model 1 was unadjusted. Model 2 was adjusted for age and sex.

Abbreviations: ePWV, estimated pulse wave velocity; CKM, cardiovascular–kidney–metabolic; OR, odds ratio; CI, confidence interval.
